# Equine Welfare during Exercise: An Evaluation of Breathing, Breathlessness and Bridles

**DOI:** 10.3390/ani7060041

**Published:** 2017-05-26

**Authors:** David J. Mellor, Ngaio J. Beausoleil

**Affiliations:** Animal Welfare Science and Bioethics Centre, Institute of Veterinary, Animal and Biomedical Science, College of Sciences, Massey University, Palmerston North 4442, New Zealand; n.j.beausoleil@massey.ac.nz

**Keywords:** horse, equine welfare, breathing during exercise, jowl angle, soft palate displacement, lower airway pathophysiology, breathlessness, dyspnoea, bit problems, bitless bridle impacts

## Abstract

**Simple Summary:**

Horses have superior athletic capabilities due largely to their exceptional cardiorespiratory responses during exercise. This has particular relevance to horses’ potential to experience breathlessness, especially when their athletic performance is reduced by impaired respiratory function. Breathlessness, incorporating three types of unpleasant experiences, has been noted as of significant animal welfare concern in other mammals. However, the potential for breathlessness to occur in horses as usually ridden wearing bitted bridles has not yet been evaluated in detail. Accordingly, key physiological responses to exercise and the consequences of impaired respiratory function are outlined. Then the physiological control of breathing and the generation of the aversive experiences of breathlessness are explained. Finally, the potential for horses with unimpaired and impaired respiratory function to experience the different types of breathlessness is evaluated. This information provides a basis for considering the circumstances in which breathlessness may have significant negative welfare impacts on horses as currently ridden wearing bitted bridles. Potential beneficial impacts on respiratory function of using bitless bridles are then discussed with emphasis on the underlying mechanisms and their relevance to breathlessness. It is noted that direct comparisons of cardiorespiratory responses to exercise in horses wearing bitless and bitted bridles are not available and it is recommended that such studies be undertaken.

**Abstract:**

Horses engaged in strenuous exercise display physiological responses that approach the upper functional limits of key organ systems, in particular their cardiorespiratory systems. Maximum athletic performance is therefore vulnerable to factors that diminish these functional capacities, and such impairment might also lead to horses experiencing unpleasant respiratory sensations, i.e., breathlessness. The aim of this review is to use existing literature on equine cardiorespiratory physiology and athletic performance to evaluate the potential for various types of breathlessness to occur in exercising horses. In addition, we investigate the influence of management factors such as rein and bit use and of respiratory pathology on the likelihood and intensity of equine breathlessness occurring during exercise. In ridden horses, rein use that reduces the jowl angle, sometimes markedly, and conditions that partially obstruct the nasopharynx and/or larynx, impair airflow in the upper respiratory tract and lead to increased flow resistance. The associated upper airway pressure changes, transmitted to the lower airways, may have pathophysiological sequelae in the alveolae, which, in their turn, may increase airflow resistance in the lower airways and impede respiratory gas exchange. Other sequelae include decreases in respiratory minute volume and worsening of the hypoxaemia, hypercapnia and acidaemia commonly observed in healthy horses during strenuous exercise. These and other factors are implicated in the potential for ridden horses to experience three forms of breathlessness—”unpleasant respiratory effort”, “air hunger” and “chest tightness”—which arise when there is a mismatch between a heightened ventilatory drive and the adequacy of the respiratory response. It is not known to what extent, if at all, such mismatches would occur in strenuously exercising horses unhampered by low jowl angles or by pathophysiological changes at any level of the respiratory tract. However, different combinations of the three types of breathlessness seem much more likely to occur when pathophysiological conditions significantly reduce maximal athletic performance. Finally, most horses exhibit clear behavioural evidence of aversion to a bit in their mouths, varying from the bit being a mild irritant to very painful. This in itself is a significant animal welfare issue that should be addressed. A further major point is the potential for bits to disrupt the maintenance of negative pressure in the oropharynx, which apparently acts to prevent the soft palate from rising and obstructing the nasopharynx. The untoward respiratory outcomes and poor athletic performance due to this and other obstructions are well established, and suggest the potential for affected animals to experience significant intensities of breathlessness. Bitless bridle use may reduce or eliminate such effects. However, direct comparisons of the cardiorespiratory dynamics and the extent of any respiratory pathophysiology in horses wearing bitted and bitless bridles have not been conducted. Such studies would be helpful in confirming, or otherwise, the claimed potential benefits of bitless bridle use.

## 1. Introduction

There is something especially engaging about seeing healthy horses running free, alert, ears forward, nostrils flared, moving across the ground with ease, displaying exceptional musculoskeletal agility, control and power. Through domestication and breeding, horses have been utilised in a wide range of human activities including competitive sporting or recreational pursuits because, compared to many other mammals, horses have superior athletic capabilities [1,2,3]. Knowledge of the integrated dynamics of whole body function underlying these capacities is extensive, and the contribution of key organ systems is well documented (e.g., [4]). Virtually every organ system is involved, directly or indirectly, but of particular interest for the present evaluation is homeostatic cardiorespiratory participation in speedy locomotion. The possibility that specific elements of physiological and pathophysiological responses to exercise may predispose horses to experience “breathlessness”, representing unpleasant respiratory sensations identified recently as a significant welfare issue in other mammals [5], is also considered here.

Note at the outset that consideration of affects, i.e., what animals experience subjectively, has a key role in contemporary animal welfare science thinking, the affects of welfare significance being those that are consciously experienced as unpleasant or pleasant rather than as hedonically neutral [6,7,8,9,10,11,12,13]. Note that animal welfare may be characterised by reference to its major features [14], which include: (1) that it is a state which is experienced subjectively by an animal; (2) that welfare-relevant experiences arise as the integrated outcomes of sensory and other neural inputs from within the animal’s body and from its environment; (3) that these sensory inputs, which reflect the animal’s internal functional states and external circumstances, are processed and interpreted by the brain according to the animal’s species-specific and individual nature, and past experience; (4) that the integrated subjective outcome of this neural processing represents the animal’s current experience (i.e., its welfare status), and this changes as the balance and character of the inputs change; and (5) that an animal’s welfare status at any one time may vary on a continuum from very bad to very good.

In short, welfare thinking focuses on what the animal experiences, the affects, considered in relation to the internal states and/or external conditions responsible for generating them [14]. Accordingly, every evaluation of an animal’s general welfare status, or specific features of it, is hypothetical to the extent that it involves making inferences about what affects may be experienced. Those inferences derive credibility from validated knowledge of the underlying systems physiology, neurophysiology and affective neuroscience, as also from the caution exercised when inferring the presence of particular affects, for example, breathlessness, thirst and pain (e.g., [5,15,16,17]). Thus, the process involves cautiously exercising scientifically informed best judgement. Here it entails using physiological knowledge to clarify whether or not particular internal states and/or external circumstances might predispose horses to experience different forms of breathlessness.

To date, no examination of breathlessness per se has been found for horses. Five questions are addressed here. First, what is the likelihood that impediments to airflow in the upper respiratory tract of exercising horses [18,19,20,21,22,23] would lead them to experience a form of breathlessness characterised as “unpleasant respiratory effort” [5]? Second, to what extent might the well-established occurrence of exercise-induced hypoxaemia, hypercapnia and/or acidaemia in horses [24,25,26,27,28,29] contribute to another form of breathlessness characterised as “air hunger” [5]? Third, might a third form of breathlessness, “chest tightness”, which is associated with inflammatory processes [5,30,31], arise independently or in association with other pathophysiological conditions [3,32,33,34,35]? Fourth, what, if any, impact might use of bitless bridles have on the ease of airflow through the upper respiratory tract [22,36,37,38,39]; the degrees of exercise-induced hypoxaemia, hypercapnia and/or acidaemia [24,25,26,27,28,29]; and the occurrence and severity of lower respiratory tract pathophysiology [3,38,40,41], and thus breathlessness? Fifth, in light of these observations, how significant might breathlessness be as an animal welfare issue in exercising horses?

This paper begins by describing key features of equine physiology and pathophysiology that are relevant to inferences about whether or not one or more of the three different forms of breathlessness may be experienced during exercise. Thus, information is provided about the horse as an obligate nasal breather, airflow capacity and resistance in the upper respiratory tract, oxygen demands in exercising horses, cardiorespiratory responses to exercise, respiratory-gas homeostasis during exercise and some pathophysiological impediments to respiratory function. The paper then continues with a brief general outline of breathlessness in mammals and an evaluation of respiratory effort, air hunger and chest tightness as they might apply to horses engaged in competitive exercise. There follows a commentary on bitted and bitless bridle use with particular reference to possible consequences for respiratory dynamics related to airflow resistance, respiratory gas homeostasis and lower airway pathophysiology in exercising horses. Finally, some implications of these and other related observations for equine welfare are discussed.

## 2. Some Key Attributes of Breathing-Related Functions in the Horse

### 2.1. Upper Airway Anatomy and Obligate Nasal Breathing

**Figure 1:**
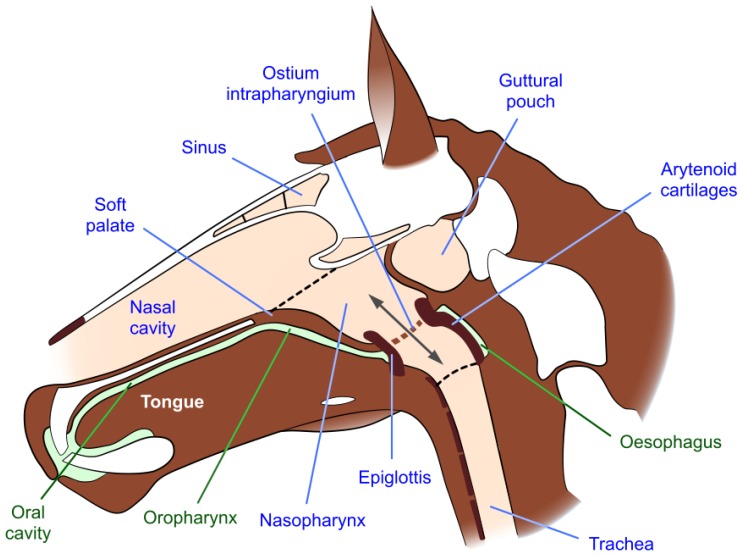
Diagram of the relationship of the soft palate and the larynx of the horse while breathing with its mouth closed (modified from Cook [22] with permission). The larynx (the “button”) fits tightly into the ostium intrapharyngium (the “buttonhole”) of the soft palate, creating an airtight seal so that air cannot enter the oropharynx (see also Figure 2). This, and closed lips, would enable a negative pressure to be maintained in the oropharynx, which would hold the soft palate against the root of the tongue thereby widening the nasopharyngeal airway [22,44]. Disengagement of the soft palate and larynx and/or loss of the lip seal would dissipate the negative pressure in the oropharynx, which would allow the soft palate to rise, vibrate with each breath, and impede nasopharyngeal airflow. The double-headed arrow indicates the directions of airflow.

**Figure 2:**
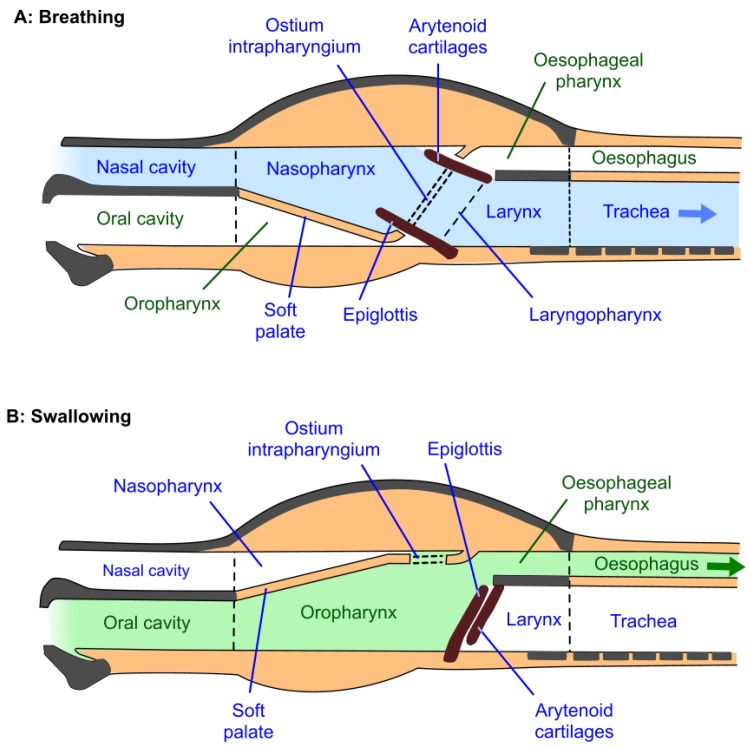
A schematic diagram of changes in pharyngeal function between the states of breathing and swallowing; derived from Cook [22] with permission. For clarity, the mouth is depicted as open and the oropharynx and oesophagus are shown as actual spaces, whereas, except during eating and drinking, the mouth is usually closed and the oropharynx and oesophagus are potential spaces only. (**A**) Breathing: Lowering of the soft palate seals off the oropharynx and enlarges the nasopharynx. The raised arytenoid cartilages close the oesophagus and open the larynx. The lowered epiglottis forms a seal with the soft palate. In this state, the larynx (the “button”) now fits snugly into the ostium intrapharyngium (the “buttonhole” of the pharynx). (**B**) Swallowing: The raised soft palate closes off the nasal cavity to prevent food and water from entering it. The arytenoid cartilages rotate down to open the oesophagus and prevent food and water from entering the larynx and trachea. Finally, the epiglottis rotates back over the arytenoid cartilages.

The upper airway of the horse includes the nostrils, nasal passages separated by the nasal septum, paired paranasal sinuses and guttural pouches, and the nasopharynx which extends from the nasal passages to the trachea [42]. The nasopharynx is located dorsal to the soft palate, which separates it from the oropharynx; the soft palate is the anatomical extension of the roof of the mouth (the hard palate) and extends from the end of the hard palate to the larynx (Figure 1 and Figure 2) [42]. It is tightly apposed to the base of the larynx, so that there is usually no communication between the nasopharynx and oropharynx, thereby obliging the horse to breathe through its nose; i.e., it is an obligate nasal breather [43]. Oral breathing can occur, but usually only when anatomical abnormalities or disruptive conditions such as dorsal displacement of the soft palate are present. The horse cannot breathe or exercise normally under these circumstances [42]. Swallowing disengages the larynx and the soft palate, allowing food and water to pass from the oropharynx into the oesophagus (Figure 2).

### 2.2. Upper Respiratory Tract Airflow Capacity and Resistance

The maximum O_2_ consumption of Thoroughbred and Standardbred racehorses at peak exertion is about 40 times greater than values at rest [42]. This is far greater than the 6–8-fold increase observed in endurance trained human athletes and the 10-fold increase seen in some other mammals [45,46]. The nasopharyngeal airflow, i.e., respiratory minute volume, required to meet these peak O_2_ demands in Thoroughbreds is approximately 1800–2000 L/min, being 25–27 fold greater than resting values of about 65–80 L/min [19,26,47]. Achieving such high airflows poses a significant physiological challenge, exacerbated when narrowing of the nasopharynx, larynx and/or trachea increases flow resistance thereby necessitating the generation of higher negative inspiratory pressures [18,20,22,48,49]. In exercising horses, the nasopharynx, larynx and trachea contribute about 95% to airflow resistance during inspiration and about 45% to it during expiration [18,29].

#### 2.2.1. Jowl Angle and Airflow Resistance

**Figure 3:**
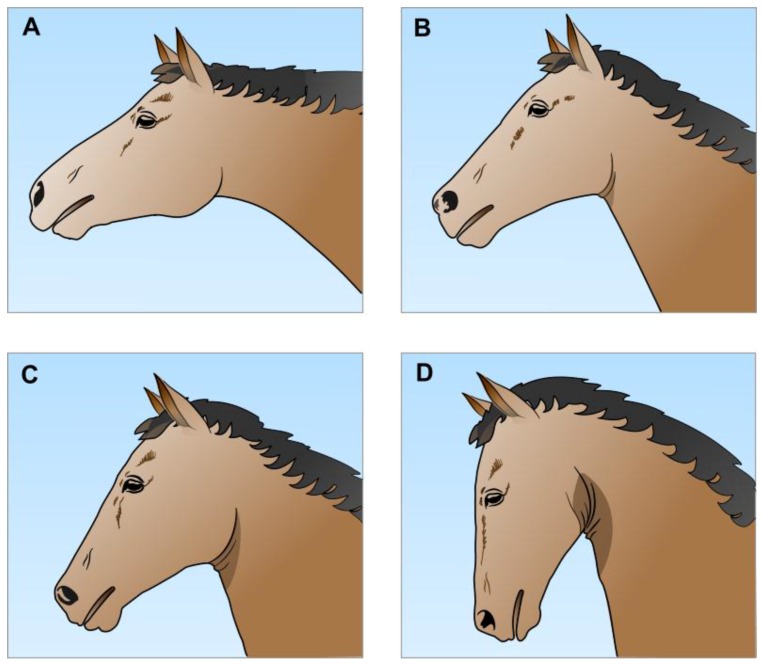
Diagrams based on photographs of horses engaged in different activities whilst wearing bitted bridles (not shown) and displaying different jowl angles: (**A**) derived from Gerring [50]; and (**B**–**D**) derived from Cook [22] who quantified nasopharyngeal areas from radiographs of the same conscious horse at different jowl angles, and compared them with the area at an extended angle. (**A**) Head-neck position of a *galloping racehorse with low rein tension* showing an extended jowl angle of about 125° observed in some animals when strenuous exercise has continued for several minutes. This jowl angle straightens and widens the diameter of the upper airway, resulting in a smooth (creaseless) “throatlatch”. However, the slightly open mouth, often seen when bitted bridles are worn [36,37,62,63], breaks the lip seal. In the remaining three examples traction was applied to the interdental space (diastema) via rein tension; this often leads to the mouth being wide open, although here it is shown as slightly open. (**B**) *A galloping racehorse.* The jowl angle is about 87°, close to the 90° resting neutral position, yet the nasopharyngeal area is reduced to about 90% of that at an extended head-neck position (e.g., A), potentially representing a significant impediment to maximal airflow. Note that the “throatlatch” is creased. (**C**) *A show jumper.* The jowl angle is about 75°, leading to a reduction in the nasopharyngeal area to about 55% of an extended position (e.g., A), representing a major hindrance to airflow. The “throatlatch” is considerably creased. Note that many show jumpers are expected to perform with significantly lower jowl angles than this [22]. (**D**) *A dressage horse*. The jowl angle is about 33° and the nasal bone is vertical to the ground. The degree of airway obstruction will be proportionately much greater than that depicted in C. Note the multiple creases in the “throatlatch”. Many dressage horses perform in a more hyperflexed position than this where the airway obstruction would be even more severe [22,59].

Head-neck position, or head carriage, is an important determinant of airflow resistance [18,22,41,49,50,51,52]. This may be expressed in terms of the jowl angle (Figure 3), i.e., the angle of intersection of the leading edge of the neck and the line of the lower jaw [22,50,51]. It has been established that the cross-sectional area of the nasopharynx decreases the closer the jowl angle approaches 33°, equivalent to the nasal bone being nearly vertical to the ground [22,23,53,54,55]. At rest the horse usually holds its head at a jowl angle of about 90°, whereas, if free to do so, when galloping it may extend its head and neck out towards jowl angles of 120–130° [22,50,51,54]. This straightens and widens the nasopharynx [22,23,53] and disproportionately reduces nasopharyngeal airflow resistance [20,51]; i.e., it *disproportionately* increases the ease of airflow in approximate compliance with Poiseuille’s Law of pressure-flow characteristics in a tube [56,57]. Such neck extension also stretches and straightens the extrathoracic trachea, making it less compliant and therefore less subject to dynamic narrowing during inspiration [18,21,22,50,54].

In contrast, when tightly reined in during exercise the horse’s nasal bone may be nearly vertical to the ground (jowl angle of about 33° or less), thereby increasing the angulation of the nasopharyngeal airway, decreasing its cross-sectional area and markedly impeding airflow at that point [22,23,55,58]. In the extreme example of the hyperflexion of the Rollkur position the cross-sectional area of the laryngeal opening is also significantly reduced [59,60]. As expected, both such reductions disproportionately increase inspiratory airflow resistance [19,48,51,52,61].

Note that various studies provide evidence that maintaining low jowl angles by rein tension can also induce or exacerbate a range of dynamic upper respiratory tract disorders during strenuous exercise (e.g., [52,54,64,65,66].

#### 2.2.2. Airflow Resistance and Disorders of the Soft Palate and Nasopharyngeal Walls

Two soft palate disorders cause narrowing of the nasopharynx and impede airflow. They are transient palatal instability (PI) and dorsal displacement of the soft palate (DDSP), the causes of which continue to be discussed [38,39,64,67,68,69,70]. One view emphasises the importance during exercise of an airtight seal between the larynx (the “button”) and the *ostium intrapharyngium* (the “buttonhole”) in the soft palate so that, when sealed, air cannot enter and dissipate the negative pressure in the oropharynx (Figure 1 and Figure 2) [22,44]. Negative oropharyngeal pressure, likely generated by swallowing [44], is said to hold the soft palate against the root of the tongue, whereas, it is argued, its dissipation allows the soft palate to bulge or balloon dorsally thereby partially obstructing the nasopharynx and larynx [44]. Such bulging, observed by dynamic endoscopy, is a distinguishing feature of PI and, moreover, appears to precede complete palatal-laryngeal disengagement and the onset of DDSP [44,71,72]. One way this seal may be broken during vigorous exercise is when the jowl angle is lower than is concordant with exercise-appropriate neck extension (Figure 3A) as a result of the horse being reined in to a marked extent (e.g., Figure 3C or D) [44,71,73]. Another proposed way is via the buccal route when aversive bit sensations are said to cause mouth opening sufficient to break the airtight lip seal [22,62,74]. A dynamic endoscopy study provides indirect support for either or both of these routes, because the wearing of a bitted bridle, as opposed to a halter, to reproduce significant neck flexion by the pressure of the bit in the mouth, was considered to be important for predisposing some horses to DDSP [71]. Potential negative impacts of the bit on respiratory function will be considered in more detail below (Section 5). 

Dynamic pharyngeal collapse may lead to partial-to-complete obstruction of the airway rostral to the glottis and marked transient increases in negative pressure with each inspiration [75]. Characterised by ventral displacement of the dorsal pharyngeal wall and/or axial displacement of the lateral walls of the pharynx, the obstruction increases in severity as the number of walls involved rises from one to three, and finally to four if PI/DDSP also occurs [75]. Likely predisposing factors for pharyngeal collapse during high-speed treadmill exercise are considered to include low jowl angles due to reign use, high negative inspiratory pressure and weakness of the stylopharyngeus muscles, normal contractions of which act to dilate the dorsal nasopharynx [51,70,71,75,76,77].

#### 2.2.3. Other Pathophysiological Impediments to Airflow in the Upper Respiratory Tract

In addition, glottal-laryngeal dysfunction can restrict airflow and increase negative inspiratory pressure, and may also be exacerbated or elicited by rein-induced low jowl angles [54,64,65,78]. Such disorders include, but are not limited to, dynamic laryngeal collapse, which may include one or more of laryngeal hemiplegia, laryngeal hemiparesis, vocal cord collapse and axial deviation of the aryepiglottic folds; other disorders include epiglottal entrapment and flaccid epiglottis [44,54,70,73,77,78]. Finally, there is potential for partial dynamic collapse of the extrathoracic trachea when negative inspiratory pressures increase in response to more rostral airway obstructions, and of the intrathoracic trachea due to compressive transmural pressure during forced expiration [18].

#### 2.2.4. Clustering of Multiple Upper Respiratory Tract Airflow Impediments

Clustering of multiple disorders has been reported in horses submitted for dynamic endoscopic examination of upper respiratory tract disorders because of suboptimal athletic performance (i.e., exercise intolerance). For example, in 471 Thoroughbred racehorses exhibiting dynamic collapse of the nasopharynx or larynx, DDSP occurred in 50% and PI in 33%, and clustered forms of dynamic collapse were apparent in 30% of the horses [44]. In 99 harness racehorses, strong associations were demonstrated between PI and intermittent DDSP, between flaccid epiglottis and dynamic laryngeal collapse due to rein-induced low jowl angles, and between axial deviation of aryepiglottic folds, PI and flaccid epiglottis [77]. The overall negative impacts of such clusters on airflow resistance might be expected to be greater than the impact of each disorder alone. 

### 2.3. Oxygen, Carbon Dioxide and Other Key Features of Respiratory Function in Galloping Horses

As already noted, the 40-fold increase in O_2_ consumption between the values at rest and at the gallop is achieved, in part, by a 25–27-fold increase in respiratory minute volume [19,26,47]. This itself is due to the combined effects of increases in respiratory frequency from 10–15 to 110–130 breaths/min and in tidal volume from 5–6 to 12–15 L [29]. Equally important for O_2_ delivery to tissues are related cardiovascular changes [46]. These include increases in heart rate from resting values of 30 beats/min, or less, to peak values of 210–230 beats/min. At a mean stroke volume of about 1.35 L, this represents a 7–8-fold increase in cardiac output from about 40 L/min or less to about 285–310 L/min.

Oxygen delivery is further enhanced by other changes. These include the effects of splenic contraction increasing the haematocrit from about 40% at rest to about 60% at the gallop and a corresponding increase in arterial blood O_2_ content from about 20 to about 30 mL O_2_/100 mL [25,46]. In addition, the O_2_ extraction by tissues increases by five-fold, or more, indicated by an equivalent widening of the carotid-arterial-to-mixed-venous difference in blood O_2_ content [25,46]. Of course, CO_2_ disposal is equally important and is referred to below.

#### 2.3.1. Oxygen and Carbon Dioxide Partial Pressures, pH and Chemoreceptor Function

The partial pressures of respiratory gases in carotid arterial blood reflect the extent to which O_2_ uptake via the lungs matches its utilisation within the body and, conversely, the extent of disposal of the CO_2_ produced by working muscle and transported to the lungs [29]. Carotid arterial pH primarily reflects whole body production of carbonic acid from CO_2_ (respiratory acidosis) and lactic acid production via anaerobic glycolysis within contracting skeletal muscle (metabolic acidosis) [25,29,79].

Changes in the arterial partial pressure of CO_2_ (PaCO_2_) and/or arterial pH, which are detected by both central and peripheral chemoreceptors, are more potent regulators of ventilation than are changes in the arterial partial pressure of oxygen (PaO_2_), which are detected only by peripheral chemoreceptors [29]. The central chemoreceptors, located in the ventral aspect of the medulla, respond to changes in the pH of cerebrospinal fluid (CSF). Rapid responses occur when changes in CSF pH are due mainly to changes in PaCO_2_ because CO_2_ diffuses freely across the blood–brain barrier [29]. In contrast, responses to changes in arterial blood pH are delayed because the barrier is relatively impermeable to H^+^ ions [29]. Accordingly, acute increases in circulating H^+^ concentrations are first detected by the peripheral chemoreceptors.

The activity of peripheral chemoreceptors, which are located in the carotid bodies and aortic arch of the horse [29], is increased by complex interactions between different degrees of hypoxaemia, hypercapnia and acidaemia [80]. Although peripheral chemoreceptor responses to CO_2_ and pH are nearly linear, their response to O_2_ evaluated separately is not, as they show enhanced activity only when the PaO_2_ decreases below about 60 mm Hg [29]. However, peripheral chemoreceptors exhibit greater sensitivity to rises in PaCO_2_ the greater the degree of the coexistent hypoxaemia [80].

#### 2.3.2. Hypoxaemia, Hypercapnia and Acidaemia in Galloping Horses

At rest, healthy high performance horses exhibit carotid PaO_2_ of 85–100 mm Hg and PaCO_2_ of 35–45 mm Hg [24,27,29,81]. Both O_2_ consumption (VO_2_) and CO_2_ production (VCO_2_) increase progressively as exercise intensity increases from resting values to the maximum [25,81]. In strenuously exercising racehorses, but not in ponies [82,83,84], hypoxaemia (PaO_2_ 60–70 mm Hg) and haemoglobin desaturation are apparent when exercise intensity exceeds 60% of the maximum O_2_ consumption capacity (VO_2max_) [25,81], whereas hypercapnia (PaCO_2_ 48–51 mm Hg) appears only when CO_2_ production is greater than 85–92% of VCO_2max_ [25]. In contrast, there is a near linear inverse relationship between carotid arterial pH and exercise intensity between about 45% and 100% of VCO_2max_ [25]. These changes in blood gas partial pressures and pH in strenuously exercising racehorses are considered to reflect limits on the exchange capacity between alveolar air and blood due to several interacting factors, including: (1) increased cardiac output reducing alveolar capillary transit time and thus impairing gas diffusion; (2) a very low PO_2_ in mixed venous blood entering the lungs, representing marked degrees of O_2_ desaturation that cannot be corrected in a single transit; and (3) the normal maximal ventilation capacity (i.e., respiratory minute volume) being inadequate due to limitations on peak tidal volume and because breathing rate is locked 1:1 with stride frequency in galloping horses [29,35,85].

Exercise-induced arterial hypoxaemia is restored to normoxaemia and hypercapnia is reversed to hypocapnia well within 5 min of healthy strenuously exercised racehorses coming to a standstill [25,29,81]. Moderate-frequency/high-tidal-volume breathing during at least the first 40–60 s after cessation of exercise restores the normoxaemia and causes a respiratory “washout” of CO_2_ [86] thereby accounting for the early appearance of hypocapnia. The rapid post-exercise changes in PaO_2_ and PaCO_2_ show that, in the face of marked reductions in VO_2_ and VCO_2_, the gas exchange capacity of the lungs greatly exceeds that required for homeostatic restoration of normoxaemia and normocapnia once exercise ceases. In contrast, there is little change in the arterial acidaemia during this period (blood pH ~7.40 at rest and ~7.13 at the gallop and shortly afterwards) [24,27]. However, immediately after exercise and for several minutes, although tidal volume progressively approaches resting values, respiratory frequency remains elevated [86,87]. This suggests that acidaemia might continue to influence respiratory activity during this period. In addition, an evaporative respiratory contribution to dissipating the exercise-induced thermal load is also likely to be involved [88,89,90]. Further salient aspects of the control of breathing are referred to below (Section 3).

Compared to healthy horses, pathophysiological conditions involving the *upper airway* that increase respiratory airflow resistance, for example, PI, DDSP, pharyngeal collapse, epiglottal entrapment and laryngeal hemiplegia [27,28,91], and experimental obstruction of the upper airway [48,91], are associated with an earlier onset of greater degrees of hypoxaemia, hypercapnia and acidaemia during exercise

### 2.4. Lower Respiratory Tract Pathophysiology and Exercise

Three *lower airway* pathophysiological conditions are considered here, namely exercise-induced pulmonary haemorrhage (EIPH), negative pressure pulmonary oedema (NPPO) and inflammatory airway disease (IAD). EIPH and NPPO represent *lower airway* conditions that are likely to be exacerbated or precipitated by increases in *upper airway* flow resistance, and IAD is considered to be equivalent to equine asthma.

#### 2.4.1. Exercise-Induced Pulmonary Haemorrhage (EIPH)

The subject of EIPH has received attention for over three decades (e.g., [3,35,40,41,48,92,93,94]. This is due to the high prevalence of the condition (70–90%) in strenuously exercising horses [3,32,33,34,35] and its negative impact on the peak athletic performance of significantly affected animals [3,94]. Although primarily occurring in Thoroughbreds, Quarter Horses and Standardbreds and during sprint racing, EIPH is also apparent among high-performance horses in cutting, barrel, roping, polo, cross-country and three-day event competitions, as well as in show jumping, hunter-jumper, steeplechase, dressage and draft horse events [35].

At present, no consensus is apparent regarding the ultimate cause of EIPH [35,38,94]. With regard to the proximate cause, markedly negative alveolar inspiratory pressure and high alveolar capillary blood pressure have been emphasised, and treatments are based on one or both of these factors [3,35]. Thus, during strenuous exercise, intra-alveolar air pressure becomes more negative and alveolar capillary blood pressure increases; this results in marked increases in the transmural pressure difference [35,48,91,95] at a time when pulmonary blood flow, equal to cardiac output, would approach maximum levels of 285–310 L/min [29,41]. The proximate cause of EIPH is therefore considered to be rupture of alveolar capillary membranes leading to extravasation of blood into the interstitial and alveolar spaces, with such ruptures occurring as a secondary consequence of the exercise-induced increase in transmural pressure exceeding the tensile strength of the capillary wall [3,35,94,96]. Note, however, that upper airway factors may be implicated in clarification of the ultimate cause of EIPH [35] because a greater alveolar transmural pressure difference is apparent in exercising horses that have partially obstructed upper airways and associated increases in upper airway resistance [48,91,95].

Grading EIPH by tracheobronchoscopic examination differentiates five levels of severity from 0 (none) to 4 (the highest) based on the amount and distribution of blood in the trachea and/or mainstream bronchi visible from the tracheal bifurcation. Although inter-observer reliability of scoring is high and it is assumed that the assigned scores reflect the severity of haemorrhage, this has not yet been established [3,94]. Nevertheless, the association of EIPH with reduced athletic performance (i.e., exercise intolerance) suggests a significant degree of pulmonary functional impairment, especially in badly affected horses (EIPH grades 3 and 4) [94]. Such severely impaired functionality is associated with filling of a proportion of alveolar air spaces with a blood/interstitial fluid mixture during a race, followed after the race by oedematous, fibrotic and angiogenic responses to haemorrhage into the alveolar interstitium [35,41,96]. The changes that occur during a race would likely act to reduce the surface area available for unimpeded respiratory gas exchange, and those that occur after the race would probably also reduce the effective surface area for gas exchange at that time and represent an impediment likely to be worsened by repeated episodes of EIPH. Although this would be expected to exacerbate the usually observed exercise-induced hypoxaemia, hypercapnia and acidaemia [24,25,27,28,29,81], there is insufficient information to clarify the extent of such effects [94]. Note, however, that the absence of evidence does not constitute evidence of absence. In this case, difficulties in prospectively identifying and acquiring horses that would be badly affected by EIPH, thereby enabling insertion of arterial catheters only into susceptible animals before exercise testing, would hinder recruitment of a sufficient number of affected animals to provide a credible basis for conclusions about impacts of EIPH on blood gas tensions and pH.

Moderate-to-strong evidence supports the following consequences of EIPH grades 3 and 4 [94]: affected horses exhibit extensive and characteristic pulmonary lesions; the condition is progressive and related to the load of racing; and affected racehorses have shorter careers, inferior competitive race performance and/or the worst race performance.

#### 2.4.2. Negative Pressure Pulmonary Oedema (NPPO)

It is speculated here that NPPO-like effects might manifest as extreme lower respiratory tract outcomes of marked upper airway obstruction due to very severe DDSP and/or pharyngeal collapse when they occur (Section 2.2.2). NPPO is a rare life threatening condition in human beings [97,98], and has also been observed in horses [99,100,101,102,103]; it develops rapidly after the onset of complete or very severe *upper airway obstruction*, for example, due to post-surgical laryngospasm following tracheal extubation. The oedema is precipitated by obstruction-induced marked increases in negative intrapleural pressure being transmitted to the alveolar interstitium, which, when combined with elevated pulmonary capillary pressure, substantially increases the transmural pressure difference [97,98,103,104]. With one reported exception [103], significant alveolar capillary bleeding is less apparent with NPPO [97,98,99,100,101,102,104] than with EIPH grades 3 and 4 [3,35,94,105], possibly because pulmonary blood flow, i.e., cardiac output, would be much lower in the absence of exercise [46]. Moderately obstructing the upper airways experimentally has partly or completely replicated EIPH pressure changes, but at lower levels [48,95,106]. This suggests that very marked nasopharyngeal obstruction may precipitate worse oedema than would less severe nasopharyngeal obstruction. Thus, in strenuously exercising horses with very severe DDSP and/or pharyngeal collapse grades 3 and 4 (Section 2.2.2), such obstruction likely occurs repeatedly with each inspiration. If so, obstruction-induced severe hypoxaemia and hypercapnia would occur immediately due to markedly impeded airflow through the upper respiratory tract, and such oedema, which hinders respiratory gas exchange via deleterious bronchiolar and alveolar effects and also reduces compliance in the *lower respiratory tract*, would likely become apparent within 1 h and persist for 24–48 h after the obstruction has been relieved [97,98,104]. There would be merit in exploring this possibility.

#### 2.4.3. Inflammatory Airway Disease (IAD)

Resembling human asthma, IAD in horses embodies a spectrum of chronic inflammatory dysfunctional effects of the lower respiratory tract [107,108] and is associated with riders’ or trainers’ subjective impressions of horses’ reduced race performance or willingness to perform in show jumping and dressage events [31]. Clinical signs include coughing and difficulty in breathing (i.e., shortness of breath), which may vary in occurrence and intensity, and are associated with variable increases in *expiratory airflow resistance* [31]. The proximate cause is considered to be irritant hypersensitivity giving rise to bronchoconstriction, bronchial oedema and mucus accumulation in bronchi [31,35]. These effects in their turn impede pulmonary gas exchange, which is evidenced by strenuously exercising horses with IAD exhibiting greater degrees of arterial hypoxaemia and a more protracted post-exercise recovery of blood gas levels than do healthy controls [109,110,111]. Clearly, this respiratory impairment will contribute to the exercise intolerance observed in IAD-affected horses [31]. Finally, in a study of 122 horses with impaired athletic performance that were diagnosed with respiratory disease, ~23% had IAD, ~16% EIPH, and ~10% had both IAD and EIPH [110].

## 3. Breathing Mechanisms That Underlie Breathlessness

Much of what follows in this section is derived from a detailed account of breathlessness in which human experience was cautiously extrapolated to other mammals [5]. The bases for this extrapolation include respiratory control mechanisms and breathing responses that appear to be common to most mammals, as well as displays of withdrawal, escape, struggling, and other aversion behaviours by non-human mammals in response to respiratory challenges that human beings find intensely unpleasant.

During normal breathing at rest, human beings usually remain unaware of sensations relating to ventilation. However, in situations where respiration is stimulated, challenged, obstructed or attended to, sensations associated with breathing rise to consciousness. Included among these is breathlessness, which is a well-recognised negative affective experience [112]. In fact, it embodies at least three qualitatively distinct sensations—respiratory effort, air hunger and chest tightness—and each of these reflects comparison by cerebral cortical processing of some combination of heightened ventilatory drive and impaired respiratory function [5]. All three major forms of breathlessness are considered to be relevant to the present evaluation.

Breathing is unusual in that its control is both automatic, via the brainstem, and voluntary, via activity of the motor cortex [113]. Automatic or reflex control of breathing is due to the collective activity of various nuclei in the medulla that stimulate pools of inspiratory motor neurons, thus influencing the frequency, strength and timing of contractions of respiratory muscles. The activity of these neurons generates the normal spontaneous pattern of breathing [114], which, in turn, is influenced by afferent inputs of four major types: (1) inputs from central and peripheral chemoreceptors responding to the PO_2_, PCO_2_, pH and other factors in arterial blood and cerebrospinal fluid; (2) afferent feedback from various respiratory structures (briefly outlined below); (3) inputs from working locomotor muscles; and (4) afferents from various higher brain centres [30,115,116]. On the other hand, voluntary control of breathing depends on efferent signals from the primary motor cortex which act to control ventilation during increased exertion and also to interrupt the normal rhythm of breathing during vocalisation and breath-holding [116,117,118].

Importantly, afferent feedback, which signals the response of the respiratory apparatus to central motor command, is also integral to both the control of breathing and the conscious awareness of respiratory sensations. Information from various receptors in the airways, lungs, respiratory muscles and chest wall, transmitted to the cortex and limbic structures, plays a key role in generating or modulating sensations of breathlessness [30,112]. The effect of such feedback on the quality and intensity of breathlessness depends on which receptors are stimulated or not stimulated, and how strongly, and on whether the source of central motor command to which the respiratory response is compared is automatic or voluntary [119].

## 4. Likely Forms of Breathlessness in Exercising Horses

In healthy horses, the voluntary and automatic components of central command to breathe would be expected to exhibit the following pattern, as is usual in healthy human beings [120,121,122]. After an initial phase of exercise dominated by voluntary command, the automatic drive to breathe would progressively increase through activation of peripheral chemoreceptors and muscle metaboreceptors [120,122]. Then, as exercise intensity approaches the aerobic threshold, inputs to the automatic drive to breathe would increase markedly, respiratory minute volume would rise steeply with increasing skeletal muscular work and the sense of the effort or work of breathing (non-aversive respiratory effort) would become increasingly intense [121,122]. Provided that a mismatch does not develop between total central command (sum of voluntary and automatic command) and the respiratory responses during exertion, unpleasant experiences of breathlessness would not be expected to occur in healthy mammals [121]. There follows a brief description of key features of *unpleasant* respiratory effort, air hunger and chest tightness and some circumstances when command-response mismatches that give rise to them may occur in exercising horses.

### 4.1. “Respiratory Effort” and High Airflow Resistance

Respiratory effort is the conscious experience of the respiratory muscle force required to achieve a necessary or desired level of ventilation, and is described in terms of the work, effort or heaviness of breathing [112,116,118]. This is usually experienced when voluntary motor command to the respiratory muscles needs to be increased to achieve the desired level of ventilation. There are two particular situations when this occurs. The first is when an increase in the depth and frequency of breathing is required during normal exercise; as noted above, this is not usually experienced as unpleasant as long as ventilation is matched with command [117]. The second, which is commonly associated with *unpleasant* respiratory effort, occurs in the presence of several pathological states when the motor command needed to elicit a given level of ventilation is greater than normal [119,121]. Thus, sensations of respiratory effort become unpleasant when the response of the ventilatory apparatus, as reported via afferent sensory inputs, especially from the respiratory muscles, is lower than expected at a given level of motor command, i.e., command and response are mismatched [116,121]. This may arise when the respiratory muscles must generate greater inspiratory or expiratory airway pressures as occurs when airflow is impeded, or when the maximum pressure-generating capacity of the respiratory system is diminished [119,121].

It is apparent from details provided above (Section 2.2) that several factors commonly observed in ridden horses would increase the likelihood that they would experience unpleasant respiratory effort of increased intensity, due primarily to increases in airflow resistance in the upper airway. The first factor is rein use that maintains jowl angles of significantly less than the resting values of about 90° (Section 2.2.1; Figure 3). Such low jowl angles very markedly restrict pharyngeal cross-sectional area near the larynx [22,23,55], and, with the extreme neck hyperflexion of the Rollkur position, reduce the cross-sectional area of the larynx as well [59].

The second factor is pathophysiological conditions that decrease the cross-sectional areas of the nasopharynx, larynx and trachea (Section 2.2.2, Section 2.2.3 and Section 2.2.4), as these structures are responsible for about 95% of inspiratory airflow resistance in healthy exercising horses [18,29]. For example, PI, DDSP, pharyngeal collapse, epiglottal entrapment and laryngeal hemiplegia [27,28] all increase airflow resistance above what are taken to be unimpeded values [48,52,91,95], and this leads to reductions in the maximum respiratory minute volume [70]. Breathlessness in the form of unpleasant respiratory effort is therefore likely to occur under these circumstances.

It is obvious that the greater the levels of exercise demanded of horses in which the cross-sectional area of the upper airway is reduced by rein use or pathophysiology, the greater would be the likelihood that they would experience unpleasant sensations of respiratory effort, and, moreover, the greater would be the likely intensity of those experiences. In addition, if both impediments occurred together, airflow resistance would be magnified, as would the anticipated intensity of the unpleasant experience of respiratory effort.

A third factor may contribute to unpleasant respiratory effort elicited by one or both of the above two factors. It relates to pathophysiological sequelae of *partial obstruction of the upper airway*, which likely increase *inspiratory* airflow resistance in the *lower respiratory tract* (Section 2.4.1 and Section 2.4.2). Identified in association with EIPH (Section 2.4.1), and, if the upper airway obstruction is very severe, possibly with NPPO-like effects (Section 2.4.2), these sequelae are: (1) physical impediments to airflow in bronchioles and bronchi due to partial or complete blockage with blood/oedema fluid; and (2) decreases in alveolar elasticity due to interstitial oedema. In addition, in horses with IAD, bronchoconstriction due to direct irritant inflammatory effects in the lower respiratory tract, including oedema, together with bronchial mucus accumulation would increase airflow resistance leading to increased *expiratory effort*, at least partly because the greater airway pressure generated during expiration tends to collapse the intrathoracic airways [31].

### 4.2. “Air Hunger” and the Chemoreceptor-Induced Drive to Breathe

Air hunger is the sensation experienced at the end of a long breath hold. Often described as “increased urge to breathe”, “shortness of breath”, “smothering” or “suffocation”, it is always reported to be unpleasant, and even moderate air hunger is more unpleasant than maximal respiratory effort [123].

Unlike respiratory effort, where the motor command is voluntary, air hunger arises mainly from a mismatch between automatic motor command and the degree of lung inflation, i.e., tidal volume [121,123,124]. Automatic drive to breathe is increased by any condition that raises PaCO_2_ and/or which reduces PaO_2_ [122]. As noted above (Section 2.3.1, Section 2.3.2 and Section 3), such changes, detected by central and peripheral chemoreceptors, provide afferent stimulation to brainstem respiratory nuclei that then alter their efferent output to respiratory muscles. During exercise, additional afferent input from metaboreceptors, which sense metabolic activity in working skeletal muscles, is transmitted to the medullary respiratory nuclei and thereby also influences ventilation [116,122]. Further details about the mechanisms underlying air hunger are available from Beausoleil and Mellor [5]. It is sufficient to re-emphasise here that air hunger occurs when there is a mismatch between automatic command and the ventilatory response to that command (i.e., limitations on tidal volume), and that the greater the mismatch the more intense will be the unpleasantness of the sensation [112]. The possible cause of tidal volume limitation is intractably high airway resistance [112] but not girth tightness, as thoracic girth circumference does not change significantly in cantering or galloping horses [85].

It is well established that healthy racehorses exhibit various combinations and degrees of arterial hypoxaemia, hypercapnia and acidaemia at different levels of exercise (Section 2.3.2), with all three conditions apparent when exercise and respiratory function are maximal [24,27,28,29,81]. The chemical drive to breathe therefore becomes progressively more intense as sustained exercise and respiratory function approach maximum levels, potentially eliciting sensations of air hunger if a mismatch develops between chemical command and ventilatory response. However, it is not known if such mismatch-induced air hunger does occur in healthy racehorses that perform athletically without apparent impediment during strenuous exercise.

In contrast, as in exercise-intolerant human beings with lung pathology [125,126], intense air hunger may contribute significantly to the impaired athletic performance often reported in horses that have pathophysiological conditions of the respiratory tract (Section 2.2.2, Section 2.2.3, Section 2.2.4, Section 2.3.2 and Section 2.4) (e.g., [3,64,94]). Likely examples include exercise-intolerant horses with partial upper airway obstruction, such as occurs with PI, DDSP, pharyngeal collapse, epiglottal entrapment and laryngeal hemiplegia [27,28,91]. In such cases, exercise induces earlier and more extreme degrees of hypoxaemia, hypercapnia and acidaemia [28,70]. Poorly performing horses having lower airway pathophysiology, for example, those affected by severe EIPH [94] and/or IAD [31] in which partial or complete blockage of bronchi as well as alveolar oedema would significantly impair pulmonary gas exchange [3,35,41,109,110,111] and likely limit tidal volume, would also be included.

### 4.3. Potential for Unpleasant “Respiratory Effort” and “Air Hunger” to Occur Simultaneously

In healthy horses, reining-in to maintain low jowl angles may lead to the occurrence of both types of breathlessness at relatively low exercise levels. As already noted, unpleasant respiratory effort may occur when the jowl angle is markedly reduced (Section 2.2.1) due to a disproportionate increase in inspiratory airflow resistance related to decreases in upper airway cross-sectional area [22,23,51,55,59]. Moreover, if any associated reduction in respiratory minute volume [70] were sufficient to impede alveolar gas exchange to an extent that caused significant hypoxaemia, hypercapnia and/or acidaemia [27,28], air hunger may be experienced as well. The likelihood of such air hunger occurring, and its intensity, would increase the greater the level of any accompanying exercise. Moreover, pathophysiological conditions, such as PI, DDSP, pharyngeal collapse, epiglottal entrapment and laryngeal hemiplegia in exercise intolerant horses [27,28,91], would also likely be accompanied both by a heightened airflow resistance as well as an increased chemical drive to breathe, giving rise, respectively, to unpleasant respiratory effort and air hunger. Thus, whether primarily due to maintenance of lower jowl angles or the presence of pathophysiological conditions, or both, both forms of breathlessness would likely be mutually reinforcing [5], and this would probably intensify the unpleasantness of the associated negative experiences especially at high levels of exercise.

### 4.4. “Chest Tightness” and Lower Respriatory Tract Inflammatroy Processes

Tightness relates specifically to the bronchoconstriction that occurs in inflammatory airway diseases such as asthma [31,80,112] or allergic bronchitis [127,128]. Tightness is understood to reflect afferent input from irritant receptors in the airways and lungs ascending to the cerebral cortex. Thus, inflammatory substances activate airway and lung C fibres and this leads to both parasympathetically controlled airway constriction and the sensation of chest tightness [129]. 

Such tightness is usually the first symptom described during an asthma attack. It often progresses to unpleasant respiratory effort, which is required to overcome airflow resistance, and because lung hyperinflation due to trapped air puts inspiratory muscles at a mechanical disadvantage [119]. In addition, air hunger may occur if this process restricts tidal volume, and/or if gas exchange is impaired [109,110,111]. It is apparent that conditions in the lower respiratory tract of horses with IAD would duplicate those that elicit chest tightness [31], and would also likely lead to simultaneous experiences of unpleasant respiratory effort and air hunger (Section 4.3).

### 4.5. Assessment of the Potential for Breathlessness to Occur in Freely Running Horses

A problem arises when considering cardiorespiratory responses to exercise and their potential relevance to unpleasant experiences of breathlessness in horses. None of the detailed information available refers to freely galloping horses unencumbered by a bridle, saddle, rider and/or equipment for making physiological measurements. It is not known, for example, whether healthy feral horses galloping voluntarily, and which are not being chased by predators or humans, would exhibit the marked hypoxaemia, hypercapnia and acidaemia observed in animals studied on treadmills during maximal exercise, nor whether they would exhibit such states when chased for extended periods. The questions of whether or not breathlessness occurs in free-running horses at full gallop, and, if so, the extent to which such animals might voluntarily modulate their exercise levels to avoid it, therefore remain unanswered.

## 5. Some Implications of Bitted and Bitless Bridle Use

### 5.1. Bits, Control of Behaviour, and Pain

Bits, fitted within the mouth, were introduced in about 2300 BC to help control horses so that they could be ridden or driven safely [1,37]. Various designs of bitted bridles enable pressure to be applied to different points on the horse’s head, especially the lips, interdental space (diastema), hard palate, tongue and lower mandible [130,131]. The bit is a potential source of considerable discomfort for the horse, as the named tissues of the mouth are extremely sensitive to mechanical stimulation. This statement is supported by clear behavioural evidence that horses find bits aversive [62,132] (see Section 5.2), as well as by convincing skeletal evidence of long-term bit-inflicted mouth injuries that would induce pain [133,134,135], features which were absent or had a much lower prevalence in feral equidae [133,134].

Experienced equestrians are well aware of mouth sensitivity as evidenced, for example, by recommendations to gently and carefully introduce the bridle whilst training young stock horses in order to avoid inducing bit shyness or aversions [136]. Even today, however, the desire to exert control over horses [132] overcomes concerns about the harm that can be done by using some bits that have been designed to apply sufficient pressure to cause significant pain-inducing soft tissue injury [131,137]. Use of such bits is increasingly regarded as inhumane [138], abusive [139] and, if tested in some jurisdictions, would likely be illegal (e.g., [140]). Of course, even simple bits that may be used gently, for example, the snaffle bit [36,37,131], when used inexpertly [132,135] or with malicious intent may cause significant injury and pain.

### 5.2. Behavioural Signs of Bit-Induced Pain and Discomfort

Distinguishing behavioural signs of bit-induced pain from other unpleasant sensations related to the irritancy of having a foreign body in contact with the tongue, gums, cheeks, lips and teeth is problematic. Nevertheless, all of these behaviours may be taken to indicate that horses displaying them find the bit aversive. Carefully observed, such behaviours have been reported to occur in various combinations in horses at rest or whilst being ridden or driven at a walk, trot, canter and gallop, and include the following: mouth slightly or widely open, persistent jaw movements, fussing with the bit; chewing, teeth grinding or holding the bit between the teeth; tongue rolling or relocation behind or over the bit; the tongue persistently moving or protruding from the mouth; excessive salivation or drooling [36,37,63,141]. Other indicative behaviours may include head shaking or tossing, and tail swishing [63,141]. Accordingly, characteristic features often seen in vigorously exercising horses ridden or driven wearing bitted bridles are an open mouth, jaw movements, dorsal relocation of the tongue and profuse salivation.

Moreover, high performance horses exhibit low, sometimes very low, jowl angles, partly open or wide open mouths, and/or head shaking or tossing at the times when reins are used to control speed, agility and direction during flat racing, steeplechase, harness racing, endurance, cross-country, show jumping, barrel racing, roping, polo and other such competitive events (Google: Youtube plus the named event, then follow all links for numerous filmed records of these equine competitions). Some of these behaviours may also be apparent during events that primarily focus on deportment, comportment and demeanour at low speed, in particular dressage and some draft horse competitions. Such behaviours likely indicate pain, especially when rein use is abrupt and/or strong. Note, however, that during races and other such events at the stages when higher speed is required, rein tension is usually released, head-neck position becomes extended (Figure 3A), and mouth opening is less apparent, for example, in horses “given their heads” or “off the bit” during the mid-to-late stages of flat races [54]. Nevertheless, direct observation reveals that some horses still have at least partly open mouths during the later stages of races, before rein tension is increased to slow them down once the race ends.

### 5.3. Mouth Behaviour of Feral Horses and Horses Wearing Bitless Bridles or Halters

The authors have carefully observed the mouth disposition of at least 150 feral horses filmed during roundups in Australia (Brumbies), France (Camargue wild horses), New Zealand (Kaimanawa wild horses) and the USA (Mustangs) (Google: Youtube, roundup, wild horse name, country, e.g., Youtube roundup Brumby Australia, then follow all links). When standing alert and when walking, trotting, cantering and galloping during roundups the mouth is invariably closed. Exceptions include vocalisation, biting, drinking and eating. Ridden horses wearing loosely-but-snugly fitted bitless bridles designed with side-pull or cross-under reins also keep their mouths closed while standing at rest or engaging in exercise ranging from walking to galloping [36,37,141,142]; so do horses wearing halters while standing in stalls or moving freely in turnout paddocks.

These observations raise two main points. First, the marked difference in mouth behaviour between animals wearing bitless bridles or halters and most of those fitted with bitted bridles reinforces conclusions regarding the potentially aversive impacts of bits [36,37,41,62,132]. Second, the striking mouth-closed (bitless) and mouth-open (bitted) difference points to a functional significance of a closed mouth that merits consideration.

### 5.4. Respiratory Functionality of a Closed Mouth in Exercising Horses

It is predicted that when healthy bit-free horses begin to walk or move at a faster pace they swallow [141]. When combined with a continuing airtight lip-seal and full engagement of the larynx and ostium intrapharyngium [44], this would contribute to generating and maintaining negative pressure in the oropharynx. After swallowing, this negative pressure would hold the soft palate firmly against the immobile root of the tongue (Figure 1) and prevent it from being sucked dorsally into the nasopharyngeal airway during inspiration [41,44], thereby maintaining a larger cross-sectional area and minimising airflow resistance as well as negative inspiratory pressure (see Section 2.2.2). In addition, it would prevent soft palatal instability and an associated increase in airflow turbulence that would otherwise occur during forceful expiratory excursions at high respiratory minute volumes [41,44]. The respiratory benefits of these effects in the exercising horse are obvious.

On the other hand, the presence of the bit itself and the mouth-opening behaviours it often elicits break the airtight lip-seal and could dissipate the negative pressure in the oral compartment [74,143]. This would destabilise the soft palate leading to its dorsal displacement into the nasopharynx during inspiration, especially during exercise [44], decreasing the cross-sectional area and thereby increasing airflow resistance as well as negative inspiratory pressure (Section 2.2.2). Palatal vibration upon expiration would also occur [41]. As noted above (Section 2.2.2), PI and DDSP are well-recognised problems in racehorses [39,41,67,68,69,70]. Interestingly, use of a crossed noseband to prevent visible mouth opening and minimise palatal dysfunction [54,144] apparently has some benefits, but the precise mechanism has not yet been clarified [54]. In addition, bit-induced tongue rolling or relocation behind or above the bit [22,67,141] has occasioned the use of tongue-ties, but with limited success [54,145] possibly because, in many cases, their application would itself precipitate loss of an airtight lip-seal.

Additionally, rein use that markedly reduces the jowl angle exacerbates these effects by decreasing the cross-sectional area of the nasopharynx near the larynx with detrimental impacts on airflow resistance and negative inspiratory pressure (Section 2.2.1) [22,23,59]. Finally, swallowing or attempting to swallow excess saliva during exercise would necessarily disengage the larynx and ostium intrapharyngium, interrupting breathing rhythm and potentially eliciting a gag or cough reflex if saliva contacts the larynx [22]. Note that repeated swallowing during high-speed treadmill studies has been observed in association with PI and/or DDSP [44,67], and that this might represent attempts to reinstate negative pressure in the oropharynx after palatal displacement has occurred [44].

### 5.5. Potential Impacts of Bitless Bridles on Breathlessness

#### 5.5.1. Bridle Type in Treadmill and Other Studies

It is important to acknowledge that cardiorespiratory responses of horses to all levels of exercise from walking to a full gallop have been extensively and rigorously investigated on treadmills and outdoor training tracks (Section 2). However, reports of treadmill studies do not routinely stipulate whether a bitted bridle or halter was used [146]. Moreover, cardiorespiratory function has not yet been compared in horses when wearing bitted bridles and halters, or bitted and bitless bridles, during the same treadmill or outdoor training track study. This hinders interpretation of results because, as indicated above, a bit in a horse’s mouth likely has negative impacts on the dynamics of airflow in the upper respiratory tract as well as functionally significant pathophysiological consequences in the lower respiratory tract. Accordingly, it is not known if such bit-induced effects have confounded some results and led to wider between-study ranges in some measured parameters.

#### 5.5.2. Bridle Type and Potential Impacts on Breathlessness

Bitless bridle use would facilitate lip-seal suction within the oral compartment that can help to maintain contiguity of the tongue and soft palate (Section 5.4), thereby minimising or avoiding palatal obstruction of the nasopharynx, especially at jowl angles of between 90° and 120–130°. This might be expected to reduce the likelihood that unpleasant experiences of respiratory effort due to increased airflow resistance within the upper respiratory tract would occur at all. Alternatively, the intensities of such experiences might be proportionately lower at all exercise levels and/or the threshold for occurrence of such experiences might be located at higher levels of exercise. Although bitless bridle use would not rule out the effects of reining-in on upper airway resistance (Section 2.2.1), users of such bridles normally aim to avoid this (e.g., [142,147]).

Impacts of bitless bridle use on the occurrence and intensity of conditions likely to induce air hunger have not been established definitively. Three possibilities present themselves. First, avoidance of bit-induced increases in nasopharyngeal airflow resistance may improve respiratory minute volume and thereby reduce the degrees of any arterial hypoxaemia, hypercapnia and acidaemia that could lead to air hunger. Such a possibility is supported by the observation that pathophysiological conditions that increase airflow resistance (e.g., see Section 2.2.2, Section 2.2.3 and Section 2.2.4) do exacerbate these three states. Second, an explicit comparison of bit and bitless bridle use may reveal a reduction in conditions likely to induce air hunger with bitless bridles, but only at lower exercise levels. This might occur if wearing a bitless bridle improved the total alveolar air-blood O_2_ and CO_2_ exchange capacities at low-levels but not at high-levels of exercise. During strenuous exercise air hunger might be more likely to occur because gas exchange capacity between alveolar air and blood is considered to be limited by the following interacting factors: (1) increased cardiac output reducing alveolar capillary transit time and thus impairing gas diffusion; (2) a very low PO_2_ in mixed venous blood entering the lungs, representing marked degrees of O_2_ desaturation that cannot be corrected in a single transit; and (3) the normal maximal ventilation capacity (i.e., respiratory minute volume) being inadequate due to limitations on peak tidal volume and because breathing rate is locked 1:1 with stride frequency in galloping horses [29,35,85]. The third and final possibility is that bit-bitless differences in circulating PO_2_, PCO_2_ and H^+^ ion concentrations may not be detectable at any exercise level.

Cook [38,41] proposed that minimising airflow resistance in the upper respiratory tract by using bitless bridles may also reduce the risk of developing pathophysiological states (e.g., EIPH) in the lower respiratory tract which are understood to impede gas exchange and reduces athletic performance (Section 2.4.1). As already noted, both unpleasant respiratory effort and air hunger are likely to be associated with the onset of severe EIPH in racehorses engaged in strenuous exercise (Section 4.1 and Section 4.2). Moreover, the combined negative affective consequences of EIPH of sufficient severity to impair athletic performance are likely to be significant. This highlights the value of exploring the extent to which bitless bridle use might beneficially reduce, or even eliminate, these untoward effects that would give rise to potentially very intense breathlessness.

## 6. Some Animal Welfare Implications

Three further questions are considered here. First, might urging racehorses to sustain maximal exercise towards the end of races increase the intensity of breathlessness they may experience? Second, what contributions might breathlessness make to fatigue in vigorously exercising horses that are healthy and fully functional or that are impaired by pathophysiological conditions? Third, how compelling is the behavioural evidence that horses find a bit or bits in their mouths aversive?

### 6.1. Breathlessness and Urging Racehorses to Sustain Maximal Exercise

Significant breathlessness of all three types can be intensely unpleasant experiences [5]. In healthy ridden or driven horses, sustained strenuous exercise generates levels of hypoxaemia, hypercapnia and/or acidaemia, which would prime the animals to experience air hunger if a mismatch were to occur between total command and the required respiratory response (Section 4.2). In addition, rein-imposed low jowl angles increase respiratory effort (Section 2.2.1) and this would also be experienced as unpleasant if a command-response mismatch were to develop during strenuous exercise (Section 4.1, Section 4.2 and Section 4.3). It is not known whether such mismatches occur in healthy fully functional racehorses that sustain apparently unimpeded high athletic performances throughout races. However, such mismatches and breathlessness seem much more likely to occur in those horses whose performance declines before races end, especially if associated with pathophysiological impediments to respiratory tract airflow and/or to alveolar gas exchange. This raises the question of whether or not whip-mediated urging by their jockeys or drivers [148,149] might lead horses to sustain high exercise levels beyond the point at which they would otherwise voluntarily slow down and thereby exacerbate the intensity of any breathlessness they may experience. The answer to this question is not clear, despite the fact that one obvious intention of whip use is to elicit and sustain maximum athletic performance [150,151].

In the absence of persistent significant pathophysiology, a rapid post-race decline in any air hunger experienced is likely to occur due to the speedy reversal of hypoxaemia and hypercapnia (but not acidaemia) during the first 2–3 min after exercise has ceased [24,25,27,29,81]. Likewise, a decline in any unpleasant respiratory effort would be expected to accompany the somewhat less rapid post-exercise return of respiratory muscle work and minute volume to near resting values [86,87]. 

The potential for bitless bridles to enhance the ease of breathing and minimise pathophysiological effects during strenuous exercise, as outlined above (Section 5), merits detailed investigation. As a first step, it is recommended that the biophysical dynamics, physiology and pathophysiology of salient features of cardiorespiratory function be rigorously compared in horses wearing bitless and bitted bridles as they exercise at different levels. Of course, a matter of direct practical interest to the horseracing industry is whether bitless bridle use can be convincingly demonstrated to achieve a better athletic performance than occurs with bitted bridle use.

### 6.2. Fatigue, Exercise Intolerance and Breathlessness

Healthy fully functional horses are said to experience *fatigue* when they can no longer sustain particular intensities of exercise and slow down [81,86], and the higher the intensity of the exercise the shorter is its duration before fatigue supervenes [152]. In the past, explanations of fatigue primarily focused on peripheral factors such as impaired energy transactions in skeletal and diaphragmatic muscles decreasing their contractile performance [66,153,154], detrimental impacts of exercise-induced hyperthermia [89], and/or other factors, all regarded as representing different forms of *homeostatic failure* [155]. In contrast, fatigue has recently been proposed to be a *protective affective experience*, generated by brain processing of exercise-related sensory inputs from the periphery, where the experience of fatigue is directed towards avoiding catastrophic failure of homeostatic mechanisms [155]. Originally referring to human athletes, this proposition may also be applicable to other mammals. If so, it would suggest that the affective experience of fatigue might have evolved to demotivate mammals from exercising at hazardously high levels, the purpose being to *safeguard their homeostatic capacity to recover* before key peripheral mechanisms that underlie strenuous locomotion fail catastrophically. It is interesting to question whether or not several centuries of breeding horses for high athletic performance might, in some respects, have exceeded such a protective role of fatigue, for example with EIPH. This question and that related to whether or not breathlessness in its various forms might reinforce or otherwise contribute to the experience of fatigue in healthy fully functioning horses during strenuous exercise, remain unresolved.

The term *exercise intolerance* is usually applied to horses that exhibit lower than expected athletic performances which are credibly linked to pathophysiological conditions of the upper and/or lower respiratory tract (e.g., [24,27,107,110,111,156]). In light of the increases in upper and/or lower airway resistance, the greater degrees of hypoxaemia, hypercapnia and acidaemia, and/or the evidence of irritant hypersensitivity causing bronchoconstriction in some cases, it seems likely that one or more of the three types of breathlessness may contribute to exercise intolerance in affected animals. However, parallel experiences of fatigue might also be generated by sensory inputs related to homeostatic support for strenuous locomotion before it reaches its limit. If so, the onset of fatigue as an affective experience may be expected to occur earlier, and/or at lower exercise intensities in such horses than in those that are healthy and fully functional.

### 6.3. Behaviours that Validly Indicate Horses’ Aversion to Bits

Observing animals living freely in their natural environments can provide valuable insights into the negative impact that human impositions have had on them (e.g., [10,157,158,159,160,161,162]). Particularly informative here are the clearly identifiable mouth behaviours indicating at least discomfort and at worst pain in most healthy horses wearing bitted bridles (Section 5.2) and their absence in feral horses and horses wearing loosely-but-snugly fitted bitless bridles or halters (Section 5.3). However, it is apparent that these behaviours are not well recognised by the majority of equestrians as indicating that horses find bits aversive. It is well established that persons accustomed to a particular spectrum of behaviours, which are almost always present, often do not recognise some of them as indicating a welfare problem. Two non-equine examples illustrate this. The first relates to dairy farmers who, prior to training, underestimated the prevalence of lameness in their herds because they considered all but the most extreme lameness gaits to be normal [163]. The second example refers to 58% of dog owners whose animals had clear clinical signs of brachycephalic obstructive airway syndrome and who reported that the dogs did not have “a breathing problem” [164]. It is suggested here, as briefly noted elsewhere [39,62], that a similar form of non-recognition of clear behavioural evidence of horses’ aversion to bits in their mouths (Section 5.2) arises because the indicative behaviours have been and are observed so commonly that, except in more extreme cases, they are considered to be normal. Once recognised, however, these behaviours cannot go “unseen”, nor should their welfare implications be ignored.

## 7. Conclusions 

It is apparent that horses engaged in strenuous exercise display physiological responses that approach the upper functional limits of key organ systems, in particular their cardiorespiratory systems. Maximum athletic performance is therefore vulnerable to factors that diminish these functional capacities. In ridden horses with bits in their mouths, rein use that reduces the jowl angle, sometimes markedly, and conditions that partially obstruct the nasopharynx and larynx, have been highlighted as factors that impair airflow in the upper respiratory tract and lead to increased flow resistance. The associated upper airway pressure changes, transmitted to the lower airways, may elicit pathophysiological sequelae in the alveolae, which, in their turn, may increase airflow resistance and impede respiratory gas exchange. Additional effects include decreases in respiratory minute volume and worsening of the hypoxaemia, hypercapnia and acidaemia that are usually observed in healthy horses during strenuous exercise.

These experimental observations, made in circumstances that approximate to those of horses ridden outdoors, draw attention to factors that may elicit the three different forms of breathlessness. However, breathlessness of any type would only occur if there were a mismatch between the total command to breathe (voluntary plus automatic) and the required respiratory response. It is not known to what extent, if at all, such mismatches would occur in strenuously exercising horses unhampered by low jowl angles, by pathophysiology at any level of the respiratory tract, or the presence of a bit (see below). However, different combinations of the three types of breathlessness seem much more likely to occur when pathophysiological conditions significantly reduce maximal athletic performance, registered as exercise intolerance. Of course, not all horses will be affected to the same extend and in precisely the same way.

Most horses exhibit clear behavioural evidence of aversion to a bit in their mouths, varying from it being a mild irritant to very painful. This in itself is a significant animal welfare issue that should be addressed. A further major point is the potential detrimental effects of bitted bridle use on the maintenance of negative pressure in the oropharynx due to loss of the airtight lip seal and/or the laryngeal-pharyngeal seal. This can apparently contribute to the development of PI and DDSP, which, when severe, can markedly increase airflow resistance in both the upper and lower respiratory tract. These changes can have wider untoward respiratory outcomes that may lead to poor athletic performance and suggest the potential for affected animals to experience significant intensities of breathlessness. Bitless bridle use may minimise or eliminate such effects. However, direct comparisons of the cardiorespiratory dynamics and extent of any respiratory pathophysiology in horses wearing bitted and bitless bridles have not been conducted. Such studies would be helpful in confirming, or otherwise, the anticipated benefits of bitless bridle use.
